# Longitudinal reallocations of time between 24-h movement behaviours and their associations with inflammation in children and adolescents: the UP&DOWN study

**DOI:** 10.1186/s12966-023-01471-9

**Published:** 2023-06-15

**Authors:** Víctor Segura-Jiménez, Željko Pedišić, Aleš Gába, Dorothea Dumuid, Timothy Olds, Nikola Štefelová, Karel Hron, Sonia Gómez-Martínez, Ascensión Marcos, José Castro-Piñero

**Affiliations:** 1 UGC Medicina Física y Rehabilitación, Hospital de Neurotraumatología y Rehabilitación, Hospital Universitario Virgen de las Nieves, Granada, Spain; 2 Instituto de Investigación Biosanitaria ibs.GRANADA, Avda. de Madrid, 15, Granada, 18012 Spain; 3 GALENO research group, Department of Physical Education, Faculty of Education Sciences, University of Cádiz, Av. República Saharaui, 12, Puerto Real, Cádiz, 11519 Spain; 4 Biomedical Research and Innovation Institute of Cádiz (INiBICA) Research Unit, Puerta del Mar University Hospital, Cádiz, Spain; 5 Institute for Health and Sport, Victoria University, Melbourne, Australia; 6 Faculty of Physical Culture, Palacký University Olomouc, Olomouc, Czech Republic; 7 Alliance for Research in Exercise, Nutrition and Activity, Allied Health & Human Performance, University of South Australia, Adelaide, Australia; 8 Centre for Adolescent Health, Murdoch Children’s Research Institute, Melbourne, Australia; 9 Department of Mathematical Analysis and Applications of Mathematics, Faculty of Science, Palacký University Olomouc, Olomouc, Czech Republic; 10 Czech Advanced Technology and Research Institute, Palacký University, Olomouc, Czech Republic; 11 Immunonutrition Group, Department of Metabolism and Nutrition, Institute of Food Science, Technology and Nutrition (ICTAN), Spanish National Research Council (CSIC), Madrid, Spain

**Keywords:** Device-measured sedentary behaviour, Device-measured physical activity, Physical activity intensity levels, Compositional data analysis

## Abstract

**Background:**

While there is evidence that physical activity, sedentary behaviour (SB) and sleep may all be associated with modified levels of inflammatory markers in adolescents and children, associations with one movement behaviour have not always been adjusted for other movement behaviours, and few studies have considered all movement behaviours in the 24-hour day as an exposure.

**Purpose:**

The aim of the study was to explore how longitudinal reallocations of time between moderate-to-vigorous physical activity (MVPA), light physical activity (LPA), SB and sleep are associated with changes in inflammatory markers in children and adolescents.

**Methods:**

A total of 296 children/adolescents participated in a prospective cohort study with a 3-year follow-up. MVPA, LPA and SB were assessed by accelerometers. Sleep duration was assessed using the Health Behavior in School-aged Children questionnaire. Longitudinal compositional regression models were used to explore how reallocations of time between movement behaviours are associated with changes in inflammatory markers.

**Results:**

Reallocations of time from SB to sleep were associated with increases in C3 levels (difference for 60 min/d reallocation [*d*_60_] = 5.29 mg/dl; 95% confidence interval [CI] = 0.28, 10.29) and TNF-α (*d*_60_ = 1.81 mg/dl; 95% CI = 0.79, 15.41) levels. Reallocations from LPA to sleep were also associated with increases in C3 levels (*d*_60_ = 8.10 mg/dl; 95% CI = 0.79, 15.41). Reallocations from LPA to any of the remaining time-use components were associated with increases in C4 levels (*d*_60_ ranging from 2.54 to 3.63 mg/dl; *p* < 0.05), while any reallocation of time away from MVPA was associated with unfavourable changes in leptin (*d*_60_ ranging from 3088.44 to 3448.07 pg/ml; *p* < 0.05).

**Conclusions:**

Reallocations of time between 24-h movement behaviours are prospectively associated with some inflammatory markers. Reallocating time away from LPA appears to be most consistently unfavourably associated with inflammatory markers. Given that higher levels of inflammation during childhood and adolescence are associated with an increased risk of chronic diseases in adulthood, children and adolescents should be encouraged to maintain or increase the level of LPA to preserve a healthy immune system.

**Supplementary Information:**

The online version contains supplementary material available at 10.1186/s12966-023-01471-9.

## Introduction

The *allostatic load theory* [1] proposes that when an individual is subjected to repeated or chronic life stresses, both physical and psychosocial, the neuroendocrine responses, including elevated levels of inflammatory markers, may have long-term physiological consequences manifesting in chronic diseases. The presence of chronic, sub-clinical inflammation in childhood and adolescence has been associated with a higher prevalence of metabolic syndrome and diabetes [2, 3], adverse health consequences in adulthood [4], and premature mortality [5]. Among the most commonly measured inflammatory markers are complement factors C3 and C4, leptin, tumour necrosis factor alpha (TNF-α), C-reactive protein (CRP), interleukin-6 (IL-6), and adiponectin. Lifestyle factors such as physical activity (PA), sedentary behaviour (SB) and sleep duration can exacerbate or attenuate the neuroendocrine response to stressors, resulting in greater or lesser likelihood of developing a disease [6, 7].

According to the framework for Viable Integrative Research in Time-Use Epidemiology (VIRTUE) [8], these movement behaviours (PA, SB and sleep) can be considered as time-use components which occur during a fixed 24-hour time window. It is not possible to increase the time spent in any one of these behaviours without an equal and opposite decrease across the remaining behaviours. The compulsory compensations that must occur to maintain the total of 24 h may have consequences for health outcomes. For example, increasing PA may be very beneficial for health outcomes if it replaces SB, but it may not be as beneficial if it replaces sleep. Compositional isotemporal substitution model allows us to investigate the health associations of replacing one behaviour with another, while keeping the remaining behaviours constant. Studies in adults support the anti-inflammatory effects of PA [9], while isotemporal substitution analyses show that reallocating time from SB to standing or stepping reduces markers of chronic low-grade inflammation [10]. In youth, there is less scientific evidence on the associations between inflammatory markers and PA [11]. It seems that exercise and PA programs reduce inflammation in overweight and obese children [12, 13], but the effect may be due to reductions in body fat and/or increases in fitness [14]. Other studies did not find significant associations between meeting PA guidelines and CRP in children and adolescents [15] nor between any PA intensity level and low-grade inflammation [16].

SB was found to be positively associated with leptin, leptin/adiponectin ratio, CRP, and IL-6 in adult women [17] and men [18]. Only a few studies have explored the relationship in the paediatric population, finding no significant relationships between total sedentary time and inflammatory markers [19, 20]. However, TV viewing, a very common type of SB, appears to be unfavourably associated with CRP levels [21].

Sleep duration also appears to be associated with inflammatory markers. In adults, short sleep was found to be associated both cross-sectionally and longitudinally with higher levels of CRP and IL-6 [22], and sleep restriction raised CRP levels [23]. In children, one study [24] yielded mixed findings on the relationship between sleep duration and inflammatory markers (CRP, IL-4, cortisol, TNF), while other studies [25] have found significant positive associations between sleep duration variability and CRP. These cross-sectional relationships may be confounded by sleep disorders such as obstructive sleep apnoea, which are themselves linked to oxidative stress in children [26].

While there is evidence that PA, SB and sleep may all be associated with modified levels of inflammatory markers in adults, adolescents and children, associations with one movement behaviour have not always been adjusted for other movement behaviours, and few studies have considered all movement behaviours in the 24-hour day as an exposure [27–30]. This is problematic because such inadequate adjustment may lead to confounding and uncertainty about which movement behaviours are important [31]. Recent methodological papers have stressed the importance of considering time-use data, including the amounts of time spent in PA, SB and sleep, as compositional data [8, 32]. In addition, we do not know whether *change* in movement behaviours is associated with *change* in inflammatory markers, because this has not yet been explored in longitudinal studies.

The aim of the current study was, therefore, to explore how longitudinal reallocations of time between moderate-to-vigorous PA (MVPA), light PA (LPA), SB and sleep are associated with changes in inflammatory markers in children and adolescents, by using compositional data analysis.

## Methods

### Participants

The data were collected as part of the UP&DOWN study; a prospective cohort study conducted in Spain [33]. Participants in the study were children aged 6–11.9 years from schools in Cádiz and adolescents aged 12–17.9 years from schools in Madrid. The overall study sample included a total of 2225 participants. Blood sampling was performed in a randomly selected subsample including one-fourth of the overall sample (*n* = 514). Detailed characteristics of the study sample are presented in Tables 1 and 2 and Supplementary Table 1.

**Table 1:** Baseline characteristics of the study sample (*n* = 296)

	Mean	SD
Age (years)	12.82	2.4
Body height (cm)	154.51	13.3
Body weight (kg)	49.90	14.3
Body mass index *z*-score	0.62	1.09
	***n***	**%**
Girls	145	49.0
Maternal education level		
No formal education	4	1.4
Primary education	53	17.9
Secondary education	52	17.6
Technical and further education	93	31.3
University degree	94	31.8
Weight status		
Underweight	1	0.3
“Normal” weight	178	60.1
Overweight	86	29.1
Obesity	31	10.5
Pubertal development		
Stage 1	11	3.7
Stage 2	79	26.7
Stage 3	100	33.8
Stage 4	77	26.0
Stage 5	29	9.8

Abbreviations: Mean = arithmetic mean, SD = standard deviation.

**Table 2:** Changes in inflammatory markers and movement behaviours over the follow-up period

	Baseline		Follow-up		Change	*p*
	Mean	SD/CI	Mean	SD/CI		
Inflammatory markers						
C3 (mg/dl)	89.35	27.82	104.75	24.20	15.40	< 0.001
C4 (mg/dl)	19.02	8.02	22.07	7.68	3.05	< 0.001
Leptin (pg/ml)	9610.29	7202.66	5183.99	5335.78	–4426.30	< 0.001
TNF-α (pg/ml)	82.28	69.52	13.43	34.58	–68.85	< 0.001
CRP (mg/dl)	1.31	2.95	0.54	3.53	–0.77	< 0.001
Interleukin-6 (pg/ml)	30.01	33.36	12.47	57.87	–17.54	< 0.001
Adiponectin (×10^6^ pg/ml)	13.43	7.52	11.85	6.99	–1.58	< 0.001
Time-use composition (h/day)						
Sleep	9.13	9.03, 9.24	8.92	8.80, 9.02	–0.21	< 0.001
Sedentary behaviour	10.89	10.73, 11.05	11.65	11.49, 11.81	0.76	< 0.001
Light physical activity	2.93	2.85, 3.02	2.48	2.41, 2.55	–0.45	< 0.001
Moderate-to-vigorous physical activity	1.05	1.00, 1.09	0.95	0.09, 1.00	–0.10	< 0.001

Abbreviations: Mean = geometric means re-scaled to collectively sum to 24 h/day for the time-use composition and arithmetic means for all other variables, SD/CI = 95% confidence intervals for the re-scaled geometric means of time-use variables and standard deviations for all other variables, *p* = *p*-value from the paired samples *t*-test (the first pivot coordinate was used for the time-use variables).

Data were collected on two occasions: first from September 2011 to June 2012, and then from September 2013 to June 2014. For the purpose of this paper, only participants with complete data at both baseline and follow-up were included in the analysis (*n* = 296, 49.0% girls). After being informed about the purpose and methodology of the study, parents of all participants provided informed consent for their children to participate in the study. The study received approval from the Committee for Research Involving Human Subjects at the University of Cádiz, the Ethics Committee of the *Hospital Puerta de Hierro* in Madrid, and the Bioethics Committee of the Spanish National Research Council.

### Measures

#### Blood sampling

Fasting blood samples were taken at the schools early in the morning. Approximately 13.5 ml of blood were extracted from the cubital vein of each participant. Levels of CRP, C3 and C4 were assessed using turbidimetry (Olympus AU2700; Olympus UK Ltd., Watford, UK) following the same procedures of previous studies [34, 35]. Sensitivity of these assessments was 0.007 mg/L, 0.01 g/L, and 0.002 g/L for CRP, C3 and C4, respectively. Leptin, TNF-α, IL-6, and adiponectin were quantified using Multiple Analyte Profiling technology (xMAP, Luminex Corporation, Austin, Texas, USA) with Bio-Plex Human Diabetes 3-Plex Assay, Bio-Plex Pro Human Chemokine TNF-α set, Bio-Plex Pro Human Cytokine IL-6 set, and Bio-Plex Pro Human Diabetes Adiponectin Assay. Sensitivity of leptin, TNF-α, IL-6, and adiponectin measurements was 3.1 pg/mL, 6.0 pg/mL, 2.6 pg/mL, and 32.7 pg/mL, respectively.

#### Physical activity, sedentary behaviour and sleep

PA and SB were assessed for seven consecutive days using GT1M, GT3X, and GT3X + accelerometers (Actigraph, Pensacola, Florida, USA). Participants were instructed to wear the accelerometer on their lower back, underneath their clothing. The device was attached to the body with an elastic belt [36]. Participants were instructed to remove the accelerometer while sleeping and engaging in water-based activities.

At least three days (including one weekend day) with ≥ 10 valid hours per day wearing the device was the criterion for inclusion in the analysis [37]. Sixty-minute periods of zero accelerometer counts with an allowance of ≤ 2 min of < 100 counts per minute were considered as non-wearing time. Analyses were conducted on data that were previously reintegrated into 10-second epochs.

The amounts of time spent in MVPA, LPA, and SB were estimated based on the previously validated cut points for activity counts on the vertical axis [38–40]: ≥2000, 100–1999, and 0–99 counts per minute, respectively. Previous findings suggest that the data collected on the vertical axis using different Actigraph accelerometer models are comparable [41]. Cleaning and processing of accelerometer data were done using the manufacturer software Actilife v.6.6.2, Actigraph, Pensacola, Florida, USA).

We assessed sleep duration using the question: ‘‘*What time did you go to bed last night and wake up this morning?*’’ from the Health Behavior in School-aged Children (HBSC) questionnaire [42]. The amount of time spent in sleep was calculated as the difference between wake-up time and bedtime and expressed in hours. Child-reported sleeping time was shown to be valid and reliable [43, 44].

#### Covariates

An electronic scale (SECA 861, SECA, Hamburg, Germany) was used to measure weight. Height was measured in the Frankfort horizontal plane using a telescopic stature-measuring instrument (SECA 225, SECA, Hamburg, Germany). We calculated body mass index as weight/height squared (kg/m^2^) and expressed it as *z*-score (*z*BMI) [45].

The participants classified themselves in one of the five pubertal development categories suggested by Tanner & Whitehouse [46]. This was based on the visual self-inspection of the development of genitals for boys and of the development of breasts for girls.

In the analyses, we also included data on participants’ sex, age (in years), and maternal education level (no formal education, primary education, secondary education, technical and further education, university degree).

### Statistical analysis

Data analysis was conducted using version 3.4.2 of R software, (R Foundation for Statistical Computing, Vienna, Austria) and version 23 of the IBM Statistical Package for the Social Sciences (SPSS Inc., an IBM Company, Chicago, IL, USA). By using the *robCompositions* package in R [47], the 4-part time-use composition including the durations of MVPA, LPA, SB, and sleep was linearly adjusted to 24 h and expressed as a specific type of isometric log-ratio (*ilr*) coordinates [48, 49].

We then performed a set of compositional regression analyses with robust estimators [50]. Each of the inflammatory markers at follow-up was used as the outcome variable in the regression models. Differences between the follow-up and baseline *ilr* coordinates of the time-use composition were used as explanatory variables. In the analyses, we made adjustments for potential confounding, by including sex, age, maternal education level, *z*BMI, change in *z*BMI, pubertal development, baseline inflammatory marker, baseline time-use composition (expressed as *ilr* coordinates), and baseline and follow-up accelerometer wear time.

Unstandardised regression coefficients from the above-mentioned regression models were used to calculate estimated changes in the outcome variables for theoretical longitudinal reallocations of time between MVPA, LPA, SB, and sleep, according to the compositional isotemporal substitution model [51]. The estimated changes in inflammatory markers were calculated using one-to-one reallocations of 10, 30 and 60 min/day (e.g. 10 min/day from SB at baseline to MVPA at follow-up) using the mean baseline composition as a starting point. We also calculated 95% confidence intervals (CI) for the estimated changes in inflammatory markers associated with the isotemporal substitutions. The estimated change in the outcome variable was considered significant if the respective 95% CI did not cover zero.

## Results

### Characteristics of the study sample

At baseline, the participants were on average 13 years old, and their average zBMI was 0.62 (Table 1). 49% of the participants were girls, and 86.5% of the participants were in the stages 2–4 of pubertal development. At baseline, the participants spent on average most of their time in sedentary behaviour (10.89 h/day), followed by sleep (9.13 h/day), LPA (2.93 h/day), and MVPA (1.05 h/day; Table 2). Over the follow-up period, sedentary time increased on average by 45.6 min. This was compensated for by less LPA (–27 min), sleep (–12.6 min), and MVPA (–6 min).

### Isotemporal substitutions and changes in C3 complement factor levels

An increase in C3 levels was associated with theoretical reallocations of time from either SB or LPA at baseline to sleep at follow-up (Table 3). The estimated changes in C3 levels associated with these isotemporal substitutions ranged from 0.89 mg/dl (for reallocation of 10 min/day from SB to sleep) to 8.10 mg/dl (for reallocation of 60 min/day from LPA to sleep). Theoretical reallocations in the opposite direction (i.e. from sleep at baseline to either SB or LPA at follow-up) were associated with a decrease in C3 levels (*p* < 0.05 for all). No significant associations with changes in C3 levels were found for other isotemporal substitutions (*p* > 0.05 for all).

**Table 3:** Estimated changes in C3 complement factor levels (mg/dl) associated with reallocations of time between physical activity, sedentary behaviour, and sleep

Reallocation	*Δ*’ (95% confidence interval)			
	↓ Sleep	↓ SB	↓ LPA	↓ MVPA
**10 min/day**				
↑ Sleep		**0.89 (0.05, 1.73)**	**1.25 (0.15, 2.34)**	0.73 (–0.66, 2.11)
↑ SB	**–0.90 (–1.74, − 0.06)**		0.36 (–0.48, 1.20)	–0.16 (–1.51, 1.19)
↑ LPA	**–1.22 (–2.28, − 0.16)**	–0.32 (–1.12, 0.48)		–0.49 (–2.30, 1.33)
↑ MVPA	–0.72 (–1.93, 0.50)	0.19 (–0.98, 1.36)	0.54 (–1.13, 2.22)	
**30 min/day**				
↑ Sleep		**2.66 (0.15, 5.17)**	**3.85 (0.44, 7.25)**	2.25 (–2.78, 7.29)
↑ SB	**–2.71 (–5.25, − 0.17)**		1.23 (–1.42, 3.88)	–0.36 (–5.32, 4.59)
↑ LPA	**–3.61 (–6.71, − 0.50)**	–0.85 (–3.16, 1.46)		–1.26 (–7.51, 5.00)
↑ MVPA	–2.14 (–5.43, 1.16)	0.62 (–2.52, 3.76)	1.81 (–3.00, 6.61)	
**60 min/day**				
↑ Sleep		**5.29 (0.28, 10.29)**	**8.10 (0.79, 15.41)**	5.76 (–15.93, 27.46)
↑ SB	**–5.49 (–10.62, − 0.37)**		2.99 (–2.84, 8.81)	0.65 (–20.99, 22.29)
↑ LPA	**–7.07 (–13.10, − 1.04)**	–1.40 (–5.79, 2.99)		–0.92 (–24.86, 23.01)
↑ MVPA	–4.28 (–10.21, 1.64)	1.38 (–4.17, 6.93)	4.20 (–5.20, 13.60)	

Abbreviations: *Δ*’ = estimated change in C3 complement factor level for the reallocation of time from the behaviour in the column to the behaviour in the row; SB = sedentary behaviour, LPA = light physical activity, MVPA = moderate-to-vigorous physical activity. An estimated change was considered significant and marked in bold, if its 95% confidence interval did not cover zero.

### Isotemporal substitutions and changes in C4 complement factor levels

An increase in C4 levels was associated with theoretical reallocations of time from LPA at baseline to any of the remaining time-use components at follow-up (*p* < 0.05 for all; Table 4). The estimated changes in C4 levels associated with these isotemporal substitutions ranged from 0.35 mg/dl (for reallocation of 10 min/day from LPA to SB) to 3.63 mg/dl (for reallocation of 60 min/day from LPA to MVPA). Theoretical reallocations in the opposite direction (i.e. from any time-use component at baseline to LPA at follow-up) were associated with a decrease in C4 levels (*p* < 0.05 for all, except for reallocation of 60 min/day from MVPA to LPA). Other isotemporal substitutions were not found to be significantly associated with changes in C4 levels (*p* > 0.05 for all).

**Table 4:** Estimated changes in C4 complement factor levels (mg/dl) associated with reallocations of time between physical activity, sedentary behaviour, and sleep

Reallocation	*Δ*’ (95% confidence interval)			
	↓ Sleep	↓ SB	↓ LPA	↓ MVPA
**10 min/day**				
↑ Sleep		0.16 (–0.07, 0.39)	**0.51 (0.23, 0.80)**	–0.10 (–0.53, 0.33)
↑ SB	–0.17 (–0.40, 0.07)		**0.35 (0.13, 0.57)**	–0.26 (–0.69, 0.16)
↑ LPA	**–0.50 (–0.77, − 0.22)**	**–0.33 (–0.54, − 0.12)**		**–0.59 (–1.13, − 0.06)**
↑ MVPA	0.06 (–0.31, 0.44)	0.23 (–0.14, 0.60)	**0.58 (0.09, 1.07)**	
**30 min/day**				
↑ Sleep		0.49 (–0.20, 1.18)	**1.61 (0.72, 2.50)**	–0.48 (–2.06, 1.10)
↑ SB	–0.50 (–1.20, 0.20)		**1.13 (0.43, 1.83)**	–0.96 (–2.52, 0.60)
↑ LPA	**–1.44 (–2.25, − 0.62)**	**–0.93 (–1.54, − 0.32)**		**–1.90 (–3.76, − 0.03)**
↑ MVPA	0.12 (–0.91, 1.14)	0.62 (–0.36, 1.61)	**1.75 (0.37, 3.13)**	
**60 min/day**				
↑ Sleep		0.96 (–0.42, 2.33)	**3.48 (1.57, 5.39)**	–3.10 (–9.94, 3.73)
↑ SB	–1.02 (–2.43, 0.38)		**2.54 (1.01, 4.08)**	–4.04 (–10.85, 2.77)
↑ LPA	**–2.76 (–4.34, − 1.18)**	**–1.72 (–2.89, − 0.55)**		–5.78 (–13.13, 1.57)
↑ MVPA	0.06 (–1.76, 1.89)	1.11 (–0.62, 2.84)	**3.63 (0.98, 6.29)**	

Abbreviations: *Δ*’ = estimated change in C4 complement factor level for the reallocation of time from the behaviour in the column to the behaviour in the row; SB = sedentary behaviour, LPA = light physical activity, MVPA = moderate-to-vigorous physical activity. An estimated change was considered significant and marked in bold, if its 95% confidence interval did not cover zero.

### Isotemporal substitutions and changes in leptin levels

An increase in leptin levels was associated with theoretical reallocations of time from MVPA at baseline to any of the remaining time-use components at follow-up (*p* < 0.05 for all; Table 5). The estimated changes in leptin levels associated with these isotemporal substitutions ranged from 170.51 pg/ml (for reallocation of 10 min/day from MVPA to sleep) to 3448.07 pg/ml (for reallocation of 60 min/day from MVPA to LPA). Theoretical reallocations in the opposite direction (i.e. from any time-use component at baseline to MVPA at follow-up) were associated with a decrease in leptin levels (*p* < 0.05 for all, except for reallocations of 60 min/day from sleep and LPA to MVPA). No significant associations with changes in leptin levels were found for other isotemporal substitutions (*p* > 0.05 for all).

**Table 5:** Estimated changes in leptin levels (pg/ml) associated with reallocations of time between physical activity, sedentary behaviour, and sleep

Reallocation	*Δ*’ (95% confidence interval)			
	↓ Sleep	↓ SB	↓ LPA	↓ MVPA
**10 min/day**				
↑ Sleep		–49.38 (–121.67, 22.91)	–68.29 (–198.27, 61.70)	**170.51 (19.13, 321.90)**
↑ SB	49.41 (–23.16, 121.98)		–19.31 (–135.46, 96.85)	**219.49 (70.59, 368.40)**
↑ LPA	66.23 (–58.73, 191.20)	16.43 (–94.37, 127.22)		**236.32 (21.43, 451.21)**
↑ MVPA	**–141.77 (–273.14, − 10.40)**	**–191.58 (–319.94, − 63.22)**	**–210.49 (–410.97, − 10.01)**	
**30 min/day**				
↑ Sleep		–148.18 (–364.39, 68.04)	–212.38 (–620.51, 195.76)	**653.82 (97.66, 1209.98)**
↑ SB	148.42 (–70.29, 367.13)		–67.80 (–435.44, 299.84)	**798.40 (248.01, 1348.80)**
↑ LPA	193.60 (–168.68, 555.88)	41.59 (–277.11, 360.29)		**843.59 (112.24, 1574.94)**
↑ MVPA	**–365.48 (–719.35, − 11.61)**	**–517.50 (–860.76, − 174.23)**	**–581.70 (–1162.17, − 1.22)**	
**60 min/day**				
↑ Sleep		–296.85 (–727.94, 134.23)	–454.36 (–1341.54, 432.82)	**3088.44 (656.50, 5520.39)**
↑ SB	297.84 (–143.30, 738.98)		–171.94 (–980.98, 637.10)	**3370.86 (943.60, 5798.12)**
↑ LPA	375.05 (–318.83, 1068.92)	62.77 (–540.51, 666.05)		**3448.07 (714.87, 6181.27)**
↑ MVPA	–603.91 (–1230.60, 22.78)	**–916.19 (–1516.94, − 315.45)**	–1073.70 (–2225.84, 78.44)	

Abbreviations: *Δ*’ = estimated change in leptin level for the reallocation of time from the behaviour in the column to the behaviour in the row; SB = sedentary behaviour, LPA = light physical activity, MVPA = moderate-to-vigorous physical activity. An estimated change was considered significant and marked in bold, if its 95% confidence interval did not cover zero.

### Isotemporal substitutions and changes in TNF-α levels

An increase in TNF-α levels was associated with theoretical reallocations of time from SB at baseline to sleep at follow-up (*p* < 0.05 for all; Table 6). The estimated changes in TNF-α levels associated with these isotemporal substitutions ranged from 0.31 pg/ml (for 10-minute reallocations) to 1.81 pg/ml (for 60-minute reallocations). Theoretical reallocations in the opposite direction (i.e. from sleep at baseline to SB at follow-up) were associated with a decrease in TNF-α levels (*p* < 0.05 for all). No significant associations with changes in TNF-α levels were found for other isotemporal substitutions (*p* > 0.05 for all).

**Table 6:** Estimated changes in TNF-α levels (pg/ml) associated with reallocations of time between physical activity, sedentary behaviour, and sleep

Reallocation	*Δ*’ (95% confidence interval)			
	↓ Sleep	↓ SB	↓ LPA	↓ MVPA
**10 min/day**				
↑ Sleep		**0.31 (0.02, 0.59)**	0.43 (–0.05, 0.91)	0.50 (–0.30, 1.30)
↑ SB	**–0.31 (–0.60, − 0.02)**		0.12 (–0.39, 0.64)	0.19 (–0.51, 0.90)
↑ LPA	–0.42 (–0.88, 0.04)	–0.11 (–0.60, 0.38)		0.08 (–0.85, 1.01)
↑ MVPA	–0.46 (–1.16, 0.24)	–0.15 (–0.75, 0.45)	–0.03 (–0.88, 0.83)	
**30 min/day**	Sleep	SB	LPA	MVPA
↑ Sleep		**0.92 (0.06, 1.77)**	1.32 (–0.19, 2.83)	1.70 (–1.21, 4.61)
↑ SB	**–0.94 (–1.80, − 0.07)**		0.42 (–1.20, 2.04)	0.80 (–1.84, 3.43)
↑ LPA	–1.25 (–2.57, 0.08)	–0.30 (–1.72, 1.13)		0.49 (–2.76, 3.73)
↑ MVPA	–1.31 (–3.20, 0.58)	–0.36 (–1.96, 1.24)	0.05 (–2.39, 2.49)	
**60 min/day**	Sleep	SB	LPA	MVPA
↑ Sleep		**1.81 (0.10, 3.53)**	2.78 (–0.53, 6.09)	6.03 (–6.34, 18.39)
↑ SB	**–1.90 (–3.64, − 0.15)**		1.02 (–2.51, 4.55)	4.27 (–7.56, 16.09)
↑ LPA	–2.45 (–4.97, 0.06)	–0.50 (–3.21, 2.22)		3.71 (–9.11, 16.54)
↑ MVPA	–2.47 (–5.83, 0.89)	–0.52 (–3.29, 2.25)	0.44 (–4.32, 5.21)	

Abbreviations: TNF = tumour necrosis factor, *Δ*’ = estimated change in TNF-α level for the reallocation of time from the behaviour in the column to the behaviour in the row; SB = sedentary behaviour, LPA = light physical activity, MVPA = moderate-to-vigorous physical activity. Statistically significant results are highlighted in bold. An estimated change was considered significant and marked in bold, if its 95% confidence interval did not cover zero.

### Isotemporal substitutions and levels of CRP, IL-6, and adiponectin

No significant association with changes in levels of CRP, IL-6 and adiponectin was found for any of the analysed isotemporal substitutions (*p* > 0.05 for all; Supplementary Tables 1–4).

## Discussion

We found that prospective reallocations of time were associated with changes in C3, C4 and TNF-α among children and adolescents. In specific, we found that increases in C3 and TNF-α levels were associated with reallocations of time from SB to sleep. Increases in C3 levels were also associated with reallocations of time from LPA to sleep. Increases in C4 levels were associated with reallocations of time from LPA to any of the remaining time-use components, while any reallocation of time away from MVPA was associated with increases in leptin level. Theoretical reallocations of time in the opposite direction were associated with decreases in the above-mentioned inflammatory markers.

### Isotemporal substitutions and changes in C3 complement factor levels

Our finding that reallocating time from SB or LPA to sleep is associated with an increase in C3 levels might be considered somewhat unexpected. A previous study did not find a significant association between levels of complement factors and sleep duration in adolescents [52], while studies among adults found that sleep deprivation is associated with increased C3 and C4 levels [53, 54]. Interestingly, a study conducted among adults found that C3 and C4 levels decrease during the night and recover during daytime, and that sleep deprivation does not affect this pattern [55]. The effect of sleep on complement factors is likely to be complex, and it needs to be further explored.

Furthermore, although around 40% of participants in the current study were overweight/obese, the C3 values for most of them were within the “normal” range (90–180 mg/dl, according to our laboratory), indicating a good general immune health. In this sense, the magnitudes of change in C3 levels associated with time reallocations found in the current study do not seem to be large enough to increase the C3 concentrations beyond the “normal” range. It is also important to note that that a certain amount of LPA might have been misclassified as SB. The choice of intensity cut-points was shown to alter the associations between the estimated time spent in different movement behaviours and a range of health outcomes [56]. Similarly, given that the HBSC question on sleep duration asks participants about the time when they went to bed last night instead of the time they fell asleep, it may also be that some screen time (i.e. a common type of SB) in bed was misclassified as sleep time. A study found that more than 85% of adolescents use their phones in bed [57]. Taking this into account, it may be that the association we found partially reflects taking time away from screen time (rather than from sleep time). However, it may also be that the magnitude of the misclassification was too small to affect our results.

### Isotemporal substitutions and changes in C4 complement factor levels

We found that reallocating time from LPA to any other time-use component (including MVPA) is associated with an increase in C4 levels, while increasing time in LPA at the expense of any other time-use component is associated with a decrease in C4 levels. This differs from previous studies, one conducted among children aged 9–10 years [14] and another among adolescents [16], finding no statistically significant association between MVPA and C4 levels. These two studies [14, 16] were cross-sectional and conducted in somewhat smaller samples than the current study, which may explain the differences from our findings. However, it is known that C4 levels are upregulated during acute inflammation response [58]. For example, a study conducted in elite cyclists found a significant increase in C4 concentrations 10 days into a cycling race [59]. In the current study, MVPA was assessed for seven consecutive days after blood sampling was performed. However, it may also be that the magnitude of the Hawthorne effect was too small to affect our results.

From our findings, it seems that replacing SB with LPA is associated with lower C4 levels. A previous study did not find a significant association between inflammatory markers and reallocations of time from LPA to SB (or vice versa) [10]. However, it should be taken into account that the study was conducted in middle-aged adults, so their results may not be directly comparable with our findings. More studies in the paediatric population are needed to confirm our findings on the association of C4 levels with reallocations between LPA and SB.

Unlike our study, a previous did not find a significant relationship between sleep duration and C4 levels in adolescents [52]. The previous study [52] was cross-sectional, it asked about habitual sleep duration, and their sample did not include children, which may explain the differences from our findings. Yet, another study found that concentrations of CRP and IL-6 increase with increased sleep duration [60], which is in accordance with our finding for reallocations of time between sleep and LPA. However, it may be that our finding is more driven by a positive impact of LPA (rather than a negative impact of long sleep duration), given that we did not find a significant association of C4 levels with reallocations of time between sleep and SB. Importantly, for most participants C4 levels were within the “normal” range (10–40 mg/dl, according to our laboratory), and the changes in C4 associated with time reallocations would probably not increase the C3 concentrations beyond the “normal” range.

### Isotemporal substitutions and changes in leptin levels

It has recently been proposed that leptin is a mechanistic link between reduced sleep duration and higher obesity risk among children [61]. However, in a previous study conducted among adolescents, leptin levels were not found to be associated with short sleep duration [62]. We also did not find significant changes in leptin levels when time was reallocated from sleep to SB or LPA.

We found that reallocating time to MVPA from any other time-use component is associated with a decrease in leptin levels. A previous study analysed the relationship between MVPA and leptin in children and adolescents, but their findings were mixed [63], possibly because they assessed MVPA using self-reports. Potentially lower reliability of self-reported MVPA (compared with the reliability of accelerometer-based estimates of MVPA in our study) may have reduced the statistical power in these studies. This could partially explain why we found that reallocating time to and from MVPA is associated with leptin levels, while several other studies did not. Additionally, unlike the previous study that was cross-sectional, our analyses were conducted on longitudinal data, which may also partially explain the differences in findings.

Previous studies using accelerometers showed that vigorous-intensity PA is negatively associated with leptin levels in European adolescents [64, 65]. Similarly, in another study, device-measured MVPA was inversely associated with leptin levels in adolescents, but leptin levels seemed to be mainly affected when PA is strenuous [66]. It may, therefore, be that vigorous-intensity PA is a key component of MVPA when it comes to positive effects on leptin levels.

### Isotemporal substitutions and changes in TNF-α levels

We found that reallocations of time from SB to sleep are associated with an increase in TNF-α levels at follow-up. In contrast, some previous studies have found a positive association between SB and TNF-α levels in adolescents [65, 67], but a recent study that assessed SB using accelerometers did not find this association among children [21]. While our findings seem to be in contrast with the previous findings for adolescents, it has to be taken into account that, unlike in previous studies, the association we found refers not only to a decrease in SB but also to a parallel increase in sleep duration. According to the literature [68], in children and adolescents the association between sleep duration with some health outcomes is U-shaped, indicating that too low as well as too high sleep duration may have adverse effects. It is also possible that some of the reported sleep time was actually SB (e.g. screen time), which potentially further complicates interpretation of this finding. We found no statistically significant association between reallocations of time to MVPA and changes in TNF-α levels which is in line with a recent meta-analysis of PA interventions and TNF-α levels in children and adolescents [69].

### Isotemporal substitutions and changes in CRP, adiponectin, and IL-6 levels

We did not find significant associations between reallocations of time and CRP levels. This concurs with a previous cross-sectional study in which device-measured PA was not found to be associated with CRP in children and adolescents [15] and a recent meta-analysis that did not find a significant effect of exercise on CRP levels in obese children and adolescents [70]. In addition, a recent meta-analysis did not find a significant association between sleep and CRP level in children [71] and device-measured sedentary time was not found to be associated with CRP levels [21]. However, a recent study found that reallocations of time from SB to vigorous-intensity PA are associated with lower CRP levels [20]. The discrepancy from our findings may be explained by the different statistical approach used; the previous study did not use the compositional data analysis. Given that we did not find a significant association of CRP levels with reallocations of time between SB and MVPA, it might be possible that PA needs to be of vigorous intensity to positively affect CRP levels.

We did not find a significant association with changes in adiponectin levels for any of the reallocations between MVPA, LPA, SB, and sleep. This is in accordance with previous studies in which adiponectin levels were not found to be associated with short sleep duration [62] and with reallocations of time from SB to PA [20].

In accordance with most previous studies, we did not find a significant association of IL-6 levels with any of the analysed reallocations of time between MVPA, LPA, SB, and sleep. While a study found an inverse relationship between PA energy expenditure and IL-6 level [72], several other studies did not confirm the association between PA intensity levels and IL-6 [16, 25, 65, 69]. Interestingly, in a 24-month longitudinal study conducted among pubertal boys, a significant positive association between engaging in 60 min/day of MVPA and serum IL-6 level was only found at baseline, while a longitudinal association between these variables was not found to be significant [73]. Previous studies did not find a significant relationship between IL-6 levels and sleep duration [25, 74] in the paediatric population. Also, no associations of IL-6 levels with reallocations of time from SB to LPA were found [20].

### Limitations and strengths of the study

The current study had several limitations. First, due to common issues associated with accelerometry, some time spent in MVPA, LPA, and SB may have been misclassified [56]. Second, the participants were instructed not to wear accelerometers while sleeping, and, therefore, we had to rely on self-reported sleep duration, which may have included a certain amount of time spent awake in bed. Third, our results may have been affected by residual confounding, due to unmeasured variables that may potentially be relevant. Fourth, one-for-one reallocations considered in this study may not always reflect real-world changes in behaviours over time. However, analysing them provides useful insights into possible time reallocation strategies that could be applied to achieve a desired outcome. Fifth, we proportionally redistributed the unaccounted time to all four behaviours, as in previous studies [29, 32, 51]. A recent study [75] has provided arguments for redistributing the unaccounted time to SB, LPA, and MVPA only. However, a formal statistical simulation would need to be conducted to determine which of the two approaches provides more accurate estimates and in which cases. Given that the participants were instructed to wear accelerometers only during waking hours, the average wear time in the current study can be considered as relatively high (i.e. mean ± standard deviation: 14.43 ± 1.05 h/day at baseline and 14.32 ± 1.22 at follow-up). This makes it unlikely that the adjustment of time-use variables to 24 h/day had a large impact on our findings.

Strengths of the study include: (1) a longitudinal study design; (2) a relatively large sample; (3) the use of accelerometers to assess MVPA, LPA, and SB; (4) adequately taking into account mathematical properties of time-use data, by using compositional data analysis; and (5) the inclusion of several inflammatory markers as outcome variables.

## Conclusions

Collectively, the results of the current study suggest that reallocations of time between MVPA, LPA, SB, and sleep are prospectively associated with some inflammatory markers, but not always consistently, or in the expected direction. Given that higher levels of inflammation during childhood and adolescence are associated with an increased risk of chronic diseases in adulthood, children and adolescents should be encouraged to use their time in a way that is likely to maintain a healthy immune system. To enable the provision of more specific recommendations on how to prevent and reduce inflammation, further research is needed on the optimal balance between MVPA, LPA, SB, and sleep in children and adolescents, especially in those at metabolic risk.

## Electronic supplementary material

Below is the link to the electronic supplementary material.

Supplementary Material 1

Supplementary Material 2

Supplementary Material 3

Supplementary Material 4

Supplementary Material 5

## Data Availability

The datasets generated during and/or analysed during the current study are available from the corresponding author on reasonable request.

## List of Abbreviations

MVPA: Moderate-to-vigorous physical activity
LPA: Light physical activity
SB: Sedentary behaviour
CI: Confidence interval
TNF-α: Tumour necrosis factor alpha
CRP: C-reactive protein
IL-6: Interleukin-6
PA: Physical activity
SB: Sedentary behaviour
BMI: Body mass index
