# Supplementary material for: Longitudinal reallocations of time between 24-h movement behaviours and their associations with inflammation in children and adolescents: the UP&DOWN study
Source: Int J Behav Nutr Phys Act. 2023 Jun 15;20:72. doi: 10.1186/s12966-023-01471-9 (PMC10268438; doi:10.1186/s12966-023-01471-9)
Supplement: Supplementary file 2 — Supplementary Material 2 [file 12966_2023_1471_MOESM2_ESM.pdf]

Supplementary table 1. Arithmetic means and standard deviations of time-use variables.

	Baseline		Follow-up	
	**Mean**	**SD**	**Mean**	**SD**
Sleep (h/day)	8.77	1.09	8.39	1.16
Sedentary behaviour (h/day)	10.48	1.41	10.95	1.40
Light physical activity (h/day)	2.89	0.76	2.40	0.64
Moderate-to-vigorous physical activity (h/day)	1.07	0.40	0.96	0.40

Abbreviations: Mean = arithmetic mean, SD = standard deviation, *p* = p-value from the paired samples *t*-test.
