# Supplementary material for: Longitudinal reallocations of time between 24-h movement behaviours and their associations with inflammation in children and adolescents: the UP&DOWN study
Source: Int J Behav Nutr Phys Act. 2023 Jun 15;20:72. doi: 10.1186/s12966-023-01471-9 (PMC10268438; doi:10.1186/s12966-023-01471-9)
Supplement: Supplementary file 3 — Supplementary Material 3 [file 12966_2023_1471_MOESM3_ESM.pdf]

Supplementary table 2. Estimated changes in C-reactive protein levels (mg/l) associated with reallocations of time between physical activity, sedentary behaviour, and sleep.

**Reallocation**	***Δ'* (95% confidence interval)**			
	↓ Sleep	↓ SB	↓ LPA	↓ MVPA
**10 minutes/day**				
↑ Sleep		0.01 (–0.01, 0.04)	0.01 (–0.03, 0.04)	0.00 (–0.04, 0.05)
↑ SB	–0.01 (–0.04, 0.01)		–0.01 (–0.05, 0.03)	–0.01 (–0.06, 0.04)
↑ LPA	–0.01 (–0.04, 0.02)	0.01 (–0.03, 0.05)		0.00 (–0.07, 0.06)
↑ MVPA	0.00 (–0.04, 0.04)	0.01 (–0.03, 0.06)	0.00 (–0.06, 0.06)	
**30 minutes/day**				
↑ Sleep		0.04 (–0.04, 0.13)	0.02 (–0.09, 0.12)	0.01 (–0.16, 0.17)
↑ SB	–0.04 (–0.13, 0.04)		–0.03 (–0.15, 0.09)	–0.04 (–0.22, 0.15)
↑ LPA	–0.02 (–0.11, 0.07)	0.03 (–0.08, 0.14)		–0.01 (–0.23, 0.21)
↑ MVPA	–0.01 (–0.12, 0.09)	0.03 (–0.09, 0.15)	0.00 (–0.16, 0.17)	
**60 minutes/day**				
↑ Sleep		0.09 (–0.09, 0.27)	0.03 (–0.19, 0.25)	–0.03 (–0.78, 0.72)
↑ SB	–0.09 (–0.27, 0.09)		–0.06 (–0.31, 0.20)	–0.11 (–0.90, 0.67)
↑ LPA	–0.04 (–0.21, 0.13)	0.06 (–0.15, 0.26)		–0.06 (–0.90, 0.78)
↑ MVPA	–0.03 (–0.22, 0.15)	0.06 (–0.16, 0.28)	0.00 (–0.33, 0.33)	

Abbreviations: *Δ'* = estimated change in C-reactive protein level for the reallocation of time from the behaviour in the column to the behaviour in the row; SB = sedentary behaviour, LPA = light physical activity, MVPA = moderate-to-vigorous physical activity.
