# Supplementary material for: Longitudinal reallocations of time between 24-h movement behaviours and their associations with inflammation in children and adolescents: the UP&DOWN study
Source: Int J Behav Nutr Phys Act. 2023 Jun 15;20:72. doi: 10.1186/s12966-023-01471-9 (PMC10268438; doi:10.1186/s12966-023-01471-9)
Supplement: Supplementary file 4 — Supplementary Material 4 [file 12966_2023_1471_MOESM4_ESM.pdf]

Supplementary table 3. Estimated changes in interleukin-6 levels (pg/ml) associated with reallocations of time between physical activity, sedentary behaviour and sleep.

Reallocation	*Δ'* (95% confidence interval)			
	↓ Sleep	↓ SB	↓ LPA	↓ MVPA
**10 minutes/day**				
↑ Sleep		0.05 (–0.13, 0.23)	0.26 (–0.03, 0.54)	–0.39 (–1.03, 0.25)
↑ SB	–0.05 (–0.23, 0.13)		0.21 (–0.07, 0.48)	–0.44 (–1.04, 0.16)
↑ LPA	–0.25 (–0.52, 0.03)	–0.19 (–0.45, 0.06)		–0.64 (–1.42, 0.15)
↑ MVPA	0.33 (–0.22, 0.88)	0.38 (–0.13, 0.89)	0.59 (–0.12, 1.30)	
**30 minutes/day**				
↑ Sleep		0.15 (–0.38, 0.68)	0.81 (–0.08, 1.70)	–1.49 (–3.84, 0.86)
↑ SB	–0.15 (–0.70, 0.39)		0.66 (–0.20, 1.52)	–1.64 (–3.87, 0.59)
↑ LPA	–0.71 (–1.49, 0.08)	–0.55 (–1.29, 0.19)		–2.19 (–4.95, 0.57)
↑ MVPA	0.85 (–0.63, 2.34)	1.01 (–0.34, 2.36)	1.67 (–0.33, 3.67)	
**60 minutes/day**				
↑ Sleep		0.30 (–0.77, 1.36)	1.78 (–0.17, 3.73)	–6.92 (–17.15, 3.31)
↑ SB	–0.31 (–1.41, 0.78)		1.49 (–0.40, 3.37)	–7.21 (–17.22, 2.79)
↑ LPA	–1.34 (–2.84, 0.16)	–1.02 (–2.43, 0.39)		–8.24 (–19.19, 2.72)
↑ MVPA	1.43 (–1.17, 4.03)	1.75 (–0.58, 4.08)	3.23 (–0.56, 7.02)	

Abbreviations: *Δ'* = estimated change in interleukin-6 level for the reallocation of time from the behaviour in the column to the behaviour in the row; SB = sedentary behaviour, LPA = light physical activity, MVPA = moderate-to-vigorous physical activity.
