# Supplementary material for: Longitudinal reallocations of time between 24-h movement behaviours and their associations with inflammation in children and adolescents: the UP&DOWN study
Source: Int J Behav Nutr Phys Act. 2023 Jun 15;20:72. doi: 10.1186/s12966-023-01471-9 (PMC10268438; doi:10.1186/s12966-023-01471-9)
Supplement: Supplementary file 5 — Supplementary Material 5 [file 12966_2023_1471_MOESM5_ESM.pdf]

Supplementary table 4. Estimated changes in adiponectin levels ($\times 10^6$ pg/ml) associated with reallocations of time between physical activity, sedentary behaviour and sleep.

Reallocation	*Δ'* (95% confidence interval)			
	↓ Sleep	↓ SB	↓ LPA	↓ MVPA
**10 minutes/day**				
↑ Sleep		0.14 (–0.01, 0.29)	0.12 (–0.10, 0.34)	0.10 (–0.24, 0.44)
↑ SB	–0.14 (–0.29, 0.01)		–0.02 (–0.21, 0.17)	–0.04 (–0.35, 0.28)
↑ LPA	–0.12 (–0.33, 0.09)	0.02 (–0.16, 0.21)		–0.02 (–0.44, 0.40)
↑ MVPA	–0.10 (–0.40, 0.20)	0.04 (–0.23, 0.31)	0.02 (–0.36, 0.41)	
**30 minutes/day**				
↑ Sleep		0.42 (–0.04, 0.88)	0.36 (–0.32, 1.04)	0.31 (–0.94, 1.56)
↑ SB	–0.42 (–0.89, 0.04)		–0.05 (–0.66, 0.56)	–0.10 (–1.27, 1.07)
↑ LPA	–0.36 (–0.96, 0.25)	0.08 (–0.46, 0.61)		–0.03 (–1.49, 1.43)
↑ MVPA	–0.30 (–1.10, 0.51)	0.14 (–0.59, 0.86)	0.08 (–1.02, 1.17)	
**60 minutes/day**				
↑ Sleep		0.83 (–0.08, 1.75)	0.74 (–0.73, 2.20)	0.78 (–4.58, 6.14)
↑ SB	–0.86 (–1.79, 0.08)		–0.06 (–1.41, 1.28)	–0.02 (–5.24, 5.20)
↑ LPA	–0.71 (–1.88, 0.46)	0.18 (–0.84, 1.20)		0.12 (–5.60, 5.84)
↑ MVPA	–0.59 (–2.03, 0.85)	0.29 (–0.96, 1.55)	0.20 (–1.92, 2.32)	

Abbreviations: TNF = tumour necrosis factor, *Δ'* = estimated change in adiponectin level for the reallocation of time from the behaviour in the column to the behaviour in the row; SB = sedentary behaviour, LPA = light physical activity, MVPA = moderate-to-vigorous physical activity.
